# Novel Pharmacological Targets of Post-Traumatic Stress Disorders

**DOI:** 10.3390/life13081731

**Published:** 2023-08-11

**Authors:** Donatella Marazziti, Claudia Carmassi, Gabriele Cappellato, Ilaria Chiarantini, Leonardo Massoni, Federico Mucci, Alessandro Arone, Miriam Violi, Stefania Palermo, Giovanni De Iorio, Liliana Dell’Osso

**Affiliations:** 1 Department of Clinical and Experimental Medicine, Section of Psychiatry, University of Pisa, 56100 Pisa, Italyliliana.dellosso@unipi.it (L.D.); 2 Saint Camillus International University of Health and Medical Sciences, 00131 Rome, Italy

**Keywords:** PTSD, pharmacological treatment, hypothalamic-pituitary-adrenal axis, opioids, glutamate, cannabinoids, oxytocin, microRNA

## Abstract

Post-traumatic stress disorder (PTSD) is a psychopathological condition with a heterogeneous clinical picture that is complex and challenging to treat. Its multifaceted pathophysiology still remains an unresolved question and certainly contributes to this issue. The pharmacological treatment of PTSD is mainly empirical and centered on the serotonergic system. Since the therapeutic response to prescribed drugs targeting single symptoms is generally inconsistent, there is an urgent need for novel pathogenetic hypotheses, including different mediators and pathways. This paper was conceived as a narrative review with the aim of debating the current pharmacological treatment of PTSD and further highlighting prospective targets for future drugs. The authors accessed some of the main databases of scientific literature available and selected all the papers that fulfilled the purpose of the present work. The results showed that most of the current pharmacological treatments for PTSD are symptom-based and show only partial benefits; this largely reflects the limited knowledge of its neurobiology. Growing, albeit limited, data suggests that the hypothalamic-pituitary-adrenal axis, opioids, glutamate, cannabinoids, oxytocin, neuropeptide Y, and microRNA may play a role in the development of PTSD and could be targeted for novel treatments. Indeed, recent research indicates that examining different pathways might result in the development of novel and more efficient drugs.

## 1. Introduction

Post-traumatic stress disorder (PTSD) is a psychiatric condition resulting from exposure to severe stressful events threatening one’s or others’ lives or physical integrity, experienced with intense distress, fear, and horror, and characterized by a multiplicity of symptoms [1]. Initially described following the first 19th-century railway disasters, PTSD has been mainly defined and studied in the USA after the first and second world wars, but especially among the veterans of the Vietnam War [2,3,4]. The epidemiological data on PTSD are variable, as they often reflect different and/or specific population groups; in any case, its prevalence is estimated to range between 5% and 10% [5], while being higher in the female sex [6,7,8] and in the military corps, where it may increase to 16.8% [9]. Post-traumatic stress disorder shows a wide range of potential symptoms, including affective and cognitive disturbances such as negative beliefs of the self or of the world, persistently depressed mood, anhedonia, feelings of estrangement or detachment from others, and an inability to experience positive emotions. Again, patients with PTSD may also suffer from hyperarousal, persistent psychological distress in response to triggers, an avoidance of places/situations reminiscent of the traumatic event, flashbacks and dissociative experiences, irritability, anger outbursts, and reckless or self-destructive behavior [10]. Furthermore, PTSD may be comorbid with mood, anxiety, and substance use disorders [1], and it may also be associated with high suicide rates [11]. Interestingly, between 3.6 and 25.6% of people may suffer from PTSD symptoms that, although not fully satisfying the criteria of the main diagnostic systems, generally go unnoticed in clinical care settings but would require medical attention since they may cause discomfort and lead to decreased family, social, and work maladjustment, and even depression with suicidal thoughts [12]. Although the pathophysiology of PTSD is largely unknown, the current biological hypotheses underline the key role of the brain circuitry regulating fear processes involving the amygdala, insula, hippocampus, and medial prefrontal cortex and related neurotransmitters, as well as the stress system that is the hypothalamic-pituitary adrenal axis (HPA) [13]. Not surprisingly, the pharmacological approach to PTSD is largely empirical and centered on the serotonin (5-HT) system, which constitutes the main target of current treatments. Nevertheless, it is evident that its multifaceted clinical picture would suggest that other systems and pathways are undoubtedly involved in its pathophysiology. In addition, the evidence that a traumatic event may not systematically lead to the development of PTSD or a trauma-related disorder strongly suggests that this condition results from the individual’s vulnerability, possibly on a genetic and/or epigenetic basis intertwined with environmental factors. Needless to say, there is an urgent need for tailored and targeted drugs to manage PTSD. If more recent data strengthen the involvement of the HPA axis and stress processes, preliminary evidence suggests that the opioid peptides, glutamate, cannabinoids, oxytocin, neuropeptide Y, and microRNA would be involved in PTSD pathophysiology [14,15,16,17,18]. Therefore, the aim of this narrative review was to present and comment on the state of the art of pharmacological treatment of PTSD and on some potential targets that might perhaps constitute novel avenues for innovative pharmacological strategies.

## 2. Materials and Methods

The following databases were accessed in order to research and gather data from articles that were published from 1 January 1995 to 31 December 2022: PubMed, Scopus, Embase, PsycINFO, and Google Scholar. Free text terms and MeSH headings were combined as follows: “(Post-traumatic stress disorder) AND (Neurobiology) AND (Pharmacological treatment)”. All the authors agreed to include in the review conference abstracts, posters of interest, and case reports if published in indexed journals. All the authors equally contributed to identifying potential information specific to this topic among the titles and abstracts evaluated.

## 3. Pharmacological Treatment of PTSD

As highlighted in the current guidelines, the pharmacological strategies of PTSD include, as first-line treatment, 5-HT reuptake inhibitors (SSRIs), in particular paroxetine, sertraline, fluoxetine, and the serotonin-noradrenaline reuptake inhibitor (SNRI) venlafaxine [19,20]. Although the lack of significant differences between SSRIs and SNRIs has not been brought into question, the USA Food and Drug Administration (FDA) includes only paroxetine and sertraline [21,22]. Despite the weaker evidence and the usually higher frequency of side effects, the second-line treatment includes amitriptyline and imipramine, two tricyclic antidepressants (TCAs), mirtazapine, a noradrenergic and specific serotonergic antidepressant (NaSSA), and phenelzine, a monoamine-oxidase inhibitor (IMAO) [19,21,22,23,24,25,26]. Cognitive-behavioral therapy (CBT) and eye-movement desensitization and processing (EMDR) are also considered first-line strategies or in association with pharmacological treatment [27]. In any case, the response rate to drugs is low, while dropout and non-response rates are high given the refractory nature of the disorder. Moreover, the presence of several psychiatric comorbidities, along with the heterogeneity of the symptoms and clinical pictures, may further contribute to the weak response rate. Indeed, antidepressants showed some effectiveness in reducing the depressive and anxious symptoms that are part of the clinical picture of PTSD but proved ineffective on other specific symptoms, such as hypervigilance, flashbacks, nightmares, difficulty in relationships, and substance abuse. It should also be considered that SSRIs and SNRIs show some adverse effects (sexual dysfunction, weight gain, sleep disturbances) that may further limit their use [28]. With regard to mirtazapine, the overall evidence is small, although it has been demonstrated to reduce nightmares in PTSD. Trazodone, in association with SSRIs, may lead to an improvement of the depressive symptoms comorbid with PTSD and of nightmares, although it shows no effect in monotherapy. Again, bupropion, a norepinephrine and dopamine reuptake inhibitor (NDRI), did not show any efficacy in PTSD [29,30]. 

Some antiadrenergic drugs are also used in PTSD, specifically propranolol, prazosin, guanfacine, and clonidine, which seem to decrease arousal, re-experiencing, and probably dissociative symptoms, as well as to improve sleep patterns. They are generally safe, but blood pressure and heart rate should be monitored. Again, it should be highlighted that propranolol can induce depressive symptoms or psychomotor slowdown [31,32,33,34,35]. Clonidine decreases norepinephrine release and could have greater effects on hyperarousal, while prazosin blocks the effects of norepinephrine. Some studies carried out on animal models reported that clonidine might block the consolidation of memories of the traumatic event [36], while the literature in humans is limited to only retrospective, open-label studies [37]. 

A few studies have been carried out in PTSD focusing on anticonvulsants such as lamotrigine, carbamazepine, pregabalin, gabapentin, tiagabine, topiramate, and valproate; however, they generally reported these drugs to be quite ineffective [38,39]. On the other hand, clinical experience has shown how some of these compounds, in particular valproate and carbamazepine, might lead to a decrease in impulsivity and hypervigilance by acting on emotional dysregulation. Olanzapine and risperidone, two second-generation antipsychotics (SGAs), have been preliminarily tested, and they seem quite useful for the treatment of ruminative thoughts, often present in subjects after a traumatic event. Just for this possible use, the European guidelines recommended risperidone in addition to SSRIs [19]. Benzodiazepines (BDZs) can be effective if used for short periods in order to avoid tolerance, abuse, and addiction, which are very common among PTSD subjects [40]. In conclusion, taken together, the available literature reveals that psychopharmacological interventions for PTSD are limited and have a small effect size [41].

## 4. Novel Pharmacological Targets in PTSD

As reviewed above, the drugs used in PTSD are mainly symptomatic or derived from limited biological abnormalities reported in the patients; this clearly reflects the lack of comprehensive knowledge of the pathophysiology of the disorder. Indeed, current research suggests that PTSD may result from impairments in fear extinction, an increase in generalized anxiety, and a negative bias toward perceiving threats from neutral inputs and feeling danger in secure settings that are also thought to be due to different neurochemical abnormalities [12,15,42,43,44,45]. In any case, the mechanisms underlying these altered processes are not totally understood. The latest information gathered on the involvement of the HPA axis and more recently on glutamate, opioids, cannabinoids, oxytocin, neuropeptide Y, and microRNA would suggest novel targets for future medications for PTSD that will be discussed below.

### 4.1. The Hypothalamic-Pituitary-Adrenal Axis

**Figure 1:**
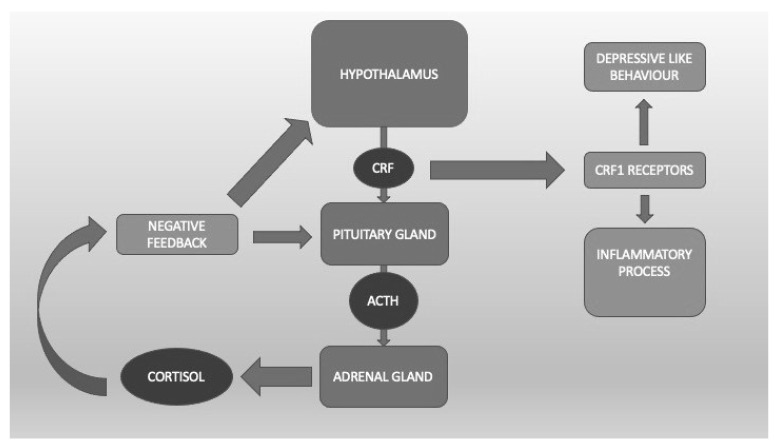
The hypothalamic-pituitary-adrenal axis. Corticotropin Releasing Factor (CRF) binds CRF1 receptors, which are linked to the inflammatory process and depressive-like behavior.

It is well known that the HPA plays an important role in the response to stress through the corticotropin-releasing factor (CRF) acting on CRF1 receptors that are present throughout the whole brain but mainly in the hypothalamus and amygdala [46] (Figure 1). Clinical data indicate that dysfunction of the stress response system, excessive CRF activity, and possible excessive stimulation of CRF1 receptors are present in a wide range of stress-related disorders such as depression, anxiety, irritable bowel syndrome [47], and, not surprisingly, PTSD. The CRF1 receptor alteration might be particularly relevant in the most severe forms of these conditions, e.g., melancholic or psychotic depression or chronic PTSD, and/or when they are accompanied by a history of early life trauma [48,49,50]. Preclinical studies demonstrate that CRF1 receptor antagonists are effective in animal models in which CRF pathways and CRF1 receptors are hyperactivated, whereas they tend to be quiescent in states of low basal CRF activity, thus indicating potentially reduced side effects in humans [51]. Symptom heterogeneity in animal models of stress and in human stress disorders might result from dysfunctions in different CRF1 receptor populations and/or functional states. Blood cortisol concentrations in PTSD patients reveal larger 24-h fluctuations than normal or depressed controls. At variance with normal states, this and the noradrenergic systems seem to stimulate each other, supporting the hypothesis of deranged negative feedback between these two systems in PTSD [17]. 

Small-molecule, orally-active CRF1 receptor antagonists may be an interesting approach for treating a range of stress-related disorders that are associated with excessive CRF1 receptor stimulation [52]. The CRF1 receptor antagonist R121919 was administered to animals for about 4 weeks, and their anxiety levels decreased without affecting their basal plasma ACTH and serum cortisol concentrations [53]. Another study reported that the administration of NBI-34041/SB723620, a novel nonapeptide acting as a CRF1 antagonist, decreased the neuroendocrine response to psychosocial stress with no effect on diurnal adrenocorticotropic hormone (ACTH) and cortisol secretion or CRF-induced ACTH and cortisol responses. According to the authors, these findings would support the notion that the CRF system is a promising target for the production of medications to treat both depression and anxiety disorders [54].

In addition, recent data showing that the neurosteroid allopregnanolone, a potent GABAergic modulator, is involved in restoring the normal functioning of the HPA axis typical of chronic stress conditions such as PTSD would indicate another potential target [55].

To summarize, these data suggesting the eventual clinical utility of the potential of CRF1 receptor modulation or HPA dampening by neurosteroids in PTSD treatment are interesting and cannot be ignored, in spite of being limited (Figure 1).

### 4.2. Opioid Peptides

The opioid peptide family includes beta-endorphins, enkephalins, and dynorphins. The first two are present in the hypothalamus, anterior and intermediate pituitary, diencephalon, pons, and locus coeruleus; the second ones are in the hypothalamus, basal ganglia, hippocampus, medullary raphe, nucleus accumbens, amygdala, and pons. Dynorphin A and B are located in the hypothalamus, dentate gyrus, periaqueductal gray, cortex, hippocampus, olfactory bulb, globus pallidus, substantia nigra, and putamen. Opioids act on three classical receptors coupled to G proteins, μ, δ, κ opioid receptors (MOR, DOR, and KOR, respectively), and on the 1/nociceptin receptor, similar to the non-classical opioid receptor [56]. It is known that the activation of each of these receptors is associated with several outcomes [57]. The MOR and DOR are linked to central anesthesia and respiratory depression, while the KOR is linked to peripheral anesthesia but not respiratory depression. The MOR is related to euphoria, while the KOR is related to dysphoria. The MOR and KOR may provoke sedation, and the only MOR has tolerance and abstinence phenomena [58]. 

Disturbances in the regulation of endogenous opioids might be involved in some symptoms of PTSD, such as freezing, stress-induced analgesia, and dissociation [59]. Therefore, potentially, opioid peptides might play a role in PTSD treatment. Some clinical evidence suggests that administration of morphine immediately after the traumatic event may reduce the likelihood of developing PTSD, although the precise mechanism of this effect is unknown [60,61]. It has been hypothesized that it may be due to an indirect decrease in noradrenergic activity or to a direct action on the interneurons of the amygdala that are critical for fear extinction processes [62]. Veterans suffering from PTSD are more likely to receive opioid prescriptions and higher or more opioid doses for the treatment of chronic pain [63]. If, from one point of view, the prescription practice contributes to a high consumption of opioids among veterans [64,65], it is well known that acute opioid administration following trauma is protective and, as already mentioned, may reduce the likelihood of developing PTSD [60,61]. Patients receiving lower doses of morphine in the first 48 h after a traumatic injury more frequently developed PTSD than patients receiving higher doses [61]. Similar results have been found in children who received morphine for burns [66]. This may be related to the effect of pain on the outcomes of PTSD. The level of pain felt at the time of a traumatic injury is predictive of the development of PTSD [67]. Another contributing factor may be the effect of morphine on long-term memory [68]. Several opioids can vary greatly in their effects, such as their ability to suppress pain, as well as in their differential risks of abuse, respiratory depression, and worsening/precipitation comorbidities with other psychological disorders [57]. Buprenorphine, a partial agonist at MOR and an antagonist at KOR, seems to show greater beneficial effects than complete agonists in the treatment of PTSD patients [69].

It is evident that the opioid system is widely involved in major PTSD symptoms and that opioid compounds may be effective. However, the abuse potential of these drugs, as reported among military groups and veterans, requires caution in their large-scale use, at least until the development of novel and less dangerous compounds.

### 4.3. Glutamate

**Figure 2:**
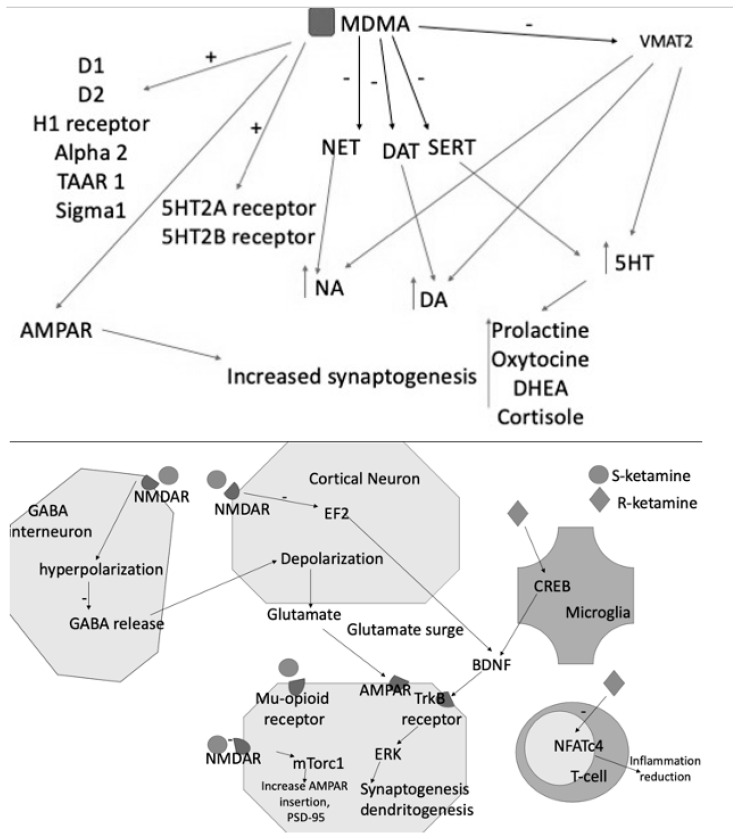
MDMA acts as an inhibitor of NET, DAT, SERT and of VMAT2, resulting in increased levels of the three monoamines DA, NE, and 5HT. The latter consequently increases the blood levels of prolactin, DHEA, oxytocin, and cortisol. MDMA also binds D1-D2 receptors and acts as an agonist of H1, 5HT2A, and B receptors. Its effects are also explained by the interaction with TAAR1, alpha2, and Sigma1. S- and R-ketamines increase glutamate release into the synaptic cleft, activating AMPAR and contributing to further synaptogenesis and dendritogenesis. S-ketamine binds NMDARs expressed in GABAergic interneurons, leading to a depolarization of cortical excitatory neurons. This depolarization causes glutamate and BDNF release, which bind to TrkB receptors. TrkB activates the mTORC1 signaling pathway, leading to the upregulation of synaptogenesis and dendritogenesis. S-ketamine binds to extrasynaptic NMDARs, disinhibiting mTORC1 signaling by deactivating eEFK2. Binding to mu-opioid receptors may facilitate antidepressant effects. R-ketamine affects microglial signaling and, by increasing BDNF release through TrkB, activates the ERK signaling pathway, resulting in synaptogenesis and dendritogenesis.

Glutamate is an excitatory neurotransmitter present in pyramidal cells and in most brain synapses, where it counterbalances the activity of the major inhibitory neurotransmitter (GABA) [70], which is one of its products through the glutamate decarboxylase. Glutamate binds to ionotropic glutamate receptors (iGluRs) that are cation-permeable ligand-gated ion channels and metabotropic glutamate receptors (mGluRs) that are G protein-coupled receptors. The activation of mGluRs and iGluRs results in distinct cellular reactions. The iGluRs are split into several functional classes, including GluD receptors (also known as delta receptors), kainate receptors, N-methyl-d-aspartate (NMDA) receptors, and -amino-3-hydroxy-5-methyl-4-isoxazolepropionic acid (AMPA) receptors [71] (Figure 2). Glutamate plays an important role in synaptic plasticity and, therefore, in cognitive functions such as learning and memory [72,73]. Animal experiments indicated that drugs capable of reducing glutamatergic transmission by inhibiting the NMDA receptors or increasing GABAergic transmission, such as neurosteroids, decrease the effects of traumatic injury [74,75]. Esketamine, a NMDA receptor inhibitor, has been marketed in some countries as an antidepressant for resistant cases with high suicidal risks [76]. Recently, esketamine has also been proposed for the treatment of PTSD, but there have been no sufficient trials on its efficacy in this condition [77,78,79]. Moreover, there is a substantial risk of abuse and addiction to this drug that is known to be higher in patients who suffer from PTSD. Recently, a multicenter experiment was carried out to examine the effectiveness of repeated intravenous ketamine doses in reducing PTSD in 158 veterans and service personnel with PTSD (*n* = 158) previously treated with antidepressants. They were randomly assigned to receive eight intravenous ketamine infusions twice a week at either a low dose (0.2 mg/kg) or a standard dose (0.5 mg/kg). Participants were examined at baseline, during therapy, and for 4 weeks following their last injection. When compared to a placebo, the usual ketamine dose dramatically reduced depression, as indicated by the MADRS. Ketamine was well tolerated, as there was no evidence of differential treatment cessation based on dose. According to this clinical investigation, the normal dose had immediate antidepressant benefits [77]. In a recent study, a group of scholars retrospectively analyzed clinical outcomes in 15 comorbid treatment-resistant depression and PTSD veterans who underwent ketamine treatment. All Veterans included in this study were administered at least six intranasal (IN)-(S)-ketamine treatments prior to switching to treatment with IV racemic ketamine. The results suggested that off-label IV (R,S)-ketamine could be considered a reasonable next step in patients who do not respond adequately to the FDA-approved IN (S)-ketamine [80]. However, even in those cases, the abuse potential of this drug is high and cannot be neglected. 3,4-methylenedioxymethamphetamine (MDMA), or ecstasy, is a popular drug of abuse with well-documented acute effects on serotonergic, dopaminergic, cholinergic, and glutamatergic systems. It is a psychedelic amphetamine that modulates AMPA receptors [81]. A study carried out on 90 PTSD patients showed a significant reduction in PTSD symptoms and severity [82]. This effect might be related to increased glutamate release and reduced parvalbumin-positive GABAergic cells in the dorsal hippocampus, as shown in rats [83]. Randomized clinical trials support the efficacy of MDMA in the treatment of PTSD. It is currently in phase III clinical trials in the United States and has been designated as a “breakthrough therapy” for the treatment of PTSD by the U.S. Food and Drug Administration (FDA) [84].

Similarly, psilocybin, a naturally occurring psychedelic compound, has been recently rediscovered for its potential therapeutic effects, particularly as a potential alternative antidepressant in MDD [85]. A preclinical study reported the anti-inflammatory effects of psilocybin, alone and in combination with eugenol, in the brain of mice with systemically induced inflammation, suggesting a potential mechanism through which psilocybin could exert its therapeutic effects in disorders associated with elevated inflammatory processes, such as PTSD [86]. However, to date, there is only one open-label pilot study, which is currently exploring the safety and efficacy of psilocybin-assisted therapy among U.S. Military veterans with severe, treatment-resistant PTSD. In particular, this study aims at investigating the combination of two psilocybin administration sessions with psychotherapy and addressing the limitations of current PTSD treatments, especially within the veteran population [87].

Taken together, the preliminary findings that glutamate modulation might constitute a possible therapeutic strategy for PTSD appear quite intriguing, even considering that esketamine is already marketed for treatment-resistant depression. Other compounds seem to show a certain degree of effectiveness that, however, needs to be substantiated in controlled clinical trials (Figure 2).

### 4.4. Cannabinoids

The cannabinoid (CBD) system, including endogenous cannabinoids, endocannabinoid production and degradation enzymes, and cannabinoid receptors, has been recently suggested to be involved in the pathophysiology of PTSD [14]. Anandamide (arachidonoyl ethanolamide) and 2-arachidonoyl glycerol (2-AG) are the first identified and well-defined endocannabinoids deriving from lipid membranes. They exert their activity through two G protein-coupled receptors called CB1 and CB2. The CB1 receptors are more abundant in the CNS than the CB2 receptors. The cortex, hippocampus, basal ganglia, and cerebellum are particularly rich in CB1 receptors, mainly on axon terminals and pre-terminal axon segments. Cortical and hippocampal CB1 receptors that are typically expressed at lower levels in glutamatergic neurons are notably concentrated in cholecystokinin (CCK)-positive interneurons. The CB1 receptors are also found in basket cells, parallel fibers, and climbing fibers in the cerebellum. Despite being identified on many neurons, CB1 receptors are also found in glial cells, and vascular tissues and microglia include this receptor. However, it is thought that some neurons express CB2, particularly in pathological circumstances such as nerve damage. A particularly intriguing property is the ability of CB2 receptor expression to rise up to 100 times following tissue injury or during inflammation. It is still unclear whether the observed increases in CB2 in the CNS are the consequence of immune cells from the periphery moving into the CNS or increased CB2 expression on CNS cells.

When required, endocannabinoids are quickly released into the extracellular space through one or two enzymatic processes (usually induced by activation of certain G protein-coupled receptors or by depolarization). The intrinsic activity of endogenous cannabinoids is different: 2-AG behaves as a high agonist at both CB1 and CB2 receptors, while anandamide shows a low and very low effect at, respectively, CB1 and CB2 receptors. Therefore, in systems with low receptor expression or when receptors connect to signaling pathways in a weak way, anandamide can counterbalance the effects of more powerful agonists. Additional endogenous substances (such as virodhamine) may increase the activity of other endocannabinoids, but the biology of these substances is less understood than that of anandamide and 2-AG. Although the effects of endocannabinoids are mostly mediated through CB1 and CB2 receptors, additional receptors, such as transient receptor potential (TRP) channels and peroxisome proliferator-activated receptor (PPAR), are supposed to mediate some endocannabinoid functions. The activation of CB1 or CB2 receptors affects cellular physiology in a variety of ways, altering gene transcription, cell motility, and synaptic function. In some circumstances, anandamide opens transient potential receptor (TRP) channels, especially TRPV1. Anandamide also activates PPAR alpha and gamma, which have a significant effect on gene transcription. It is important to keep in mind that boosting anandamide levels by blocking fatty acid amide hydrolase (FAAH) breakdown also increases concentrations of other N-acylamides that may affect PPAR [88]. 

Different studies suggest alterations of endogenous cannabinoids may be present in PTSD [89,90,91], so in recent years, a growing interest has emerged in the therapeutic use of cannabis and CBDs for the treatment of this disorder [92]. Indeed, animal studies showed that CBDs can facilitate the interruption of the consolidation of fear memories [93] and decrease anxiety through their activity on CB1 receptors, with a dose-dependent biphasic effect producing anxiolytic-like effects at low doses and an anxiogenic response at higher doses [94]. Nabilone, a synthetic cannabinoid agonist already approved by the FDA for the treatment of chemotherapy-induced nausea and vomiting, appears to be the most explored synthetic cannabinoid in the treatment of PTSD [95], as it may improve insomnia and nightmares related to trauma and reduce daytime flashbacks [96,97,98,99]. According to some studies, CBD may prevent the reconsolidation of traumatic memories by lessening trauma recall. Moreover, tetrahydrocannabinol (THC), the main psychoactive constituent of cannabis, has been reported to modify threat-related processing in PTSD patients, and it might perhaps be helpful in treating stress- and trauma-related psychopathology [100]. 

The studies on CBDs in relation to PTSD are still meager but nevertheless interesting, as they would suggest a really innovative approach to targeting some of its specific symptoms.

### 4.5. Oxytocin

**Figure 3:**
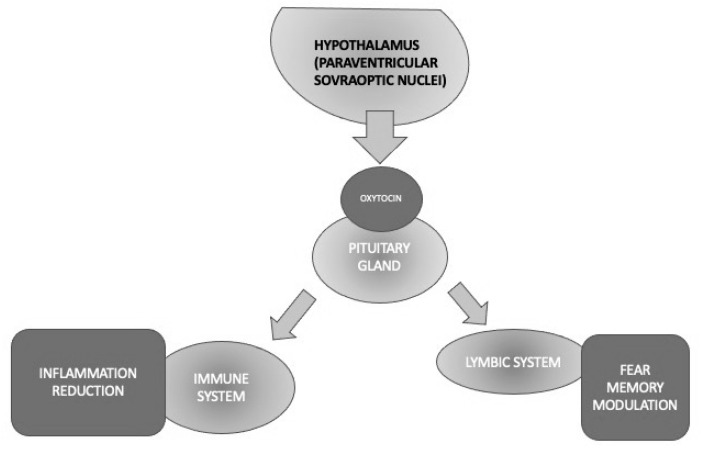
Different activities of oxytocin implicated in PTSD through its anti-inflammatory and fear memory modulation properties.

Oxytocin (OT) is a pleiotropic hormone produced in the paraventricular and supraoptic nuclei of the hypothalamus and released into the bloodstream through the posterior pituitary gland and into the brain through several pathways [99]. Oxytocin seems to promote social interactions between conspecifics, attachment, and pair-bonding, and it also exhibits anti-inflammatory properties as a powerful immune and stress system modulator, beyond its “classical” activities, such as uterine contractions during labor and milk production [45,101,102] (Figure 3). 

The first evidence that the OT might be involved in the pathophysiology of PTSD derived from studies on animals showing that exogenous OT injection reduced sympathetic responses, such as heart rate, blood pressure, and cortisol levels, and attenuated the activation of the HPA axis in threatening situations [103,104]. Such findings highlighted that these effects of OT were due to decreased activation of the central amygdala. Data in humans reported a relationship between traumatic events and/or PTSD following severe childhood abuse and reduced endogenous OT concentrations [105,106,107,108]. Genetic association studies revealed that some OTR gene polymorphisms might be associated with an increased risk of experiencing traumatic events and/or developing psychiatric disorders, such as PTSD, anxiety, and depression, while other OTR gene polymorphisms may have a protective role. The most studied polymorphism, namely that of the OXTR rs53576 GG genotype, was associated with insecure attachments, poor response to social support, emotional dysregulation, and less resilience to stress, all factors that are linked to a greater vulnerability to psychiatric disorders related to traumatic experiences [109]. The direct measurements of plasma OT levels in patients with PTSD are few and controversial while reporting controversial findings with women generally reported to show higher concentrations than men [110,111]. The reported differences could be explained by the fact that sex hormones affect OT release and its receptor expression.

Data show that more than 95% of the OT given through the nasal cavity is transported in the brain and modulates amygdala and hippocampal responses. A study involving healthy men revealed improved communication between the hippocampus and amygdala, as well a reduced amygdala activity in both healthy and psychiatric individuals, according to a meta-analysis of 66 fMRI investigations [39]. Using this route of administration, clinical trials are currently being undertaken to assess OT’s potential as a treatment for different neuropsychiatric conditions [112,113,114,115,116]. It is relevant for the pathophysiology of PTSD that the fear memory or stress-related memory is modulated by OT [117], and it is related to an asymmetry of dentate gyrus (DG)/CA3 activity. This alteration might be restored by chemogenetic inactivation of OT receptor-positive interneurons localized in the hilus (Oxtr-HI) and by inactivation of dorsal-hippocampal efferents to the caudal lateral septum [117]. 

Therefore, OT has been suggested as a promising pharmacological agent for drug-enhanced psychotherapy in PTSD because of its anxiolytic and prosocial properties [118]. These authors demonstrated that the administration of OT dampened subjective anxiety and nervousness in PTSD patients and that aberrant functional connectivity models in PTSD patients were normalized to levels similar to those in controls exposed to trauma. This suggests that OT has the potential to decrease fear expression and improve response to treatment in PTSD patients. In line with the notion of muffled fear expression, patients with PTSD showed a decrease in anxiety and nervousness after OT but no mood changes. According to a different study, participants in the PTSD group who received a single dosage of intranasal OT performed better on the most challenging working memory test condition when compared to those who received a placebo [119]. Another study showed that intranasal OT might be a tool to decrease the salience network’s hyperconnectivity described in the amygdala and anterior insula (AI) of PTSD patients. In response to fear-inducing stimuli, OT reduced connectivity between the amygdala and AI. However, unlike women, men did not experience this reduction in connectivity between the left amygdala and the left AI. On the other hand, men but not women experienced a reduction in the connectivity between the right amygdala and the right AI. The findings imply that intranasal OT modifies danger salience in people who have experienced childhood trauma and that these effects differ depending on gender and hemisphere [120]. On the contrary, a recent trial involving 220 women led to negative results [121].

To sum up, OT is a hormone that has been well known for decades for exerting the modulation of a variety of functions that are core elements of PTSD, such as stress and fear/anxiety responses. The exact role of OT in stress processes is still unclear; however, it has been reported to decrease the response of the HPA axis in rodents and primates. Other physiological OT activities, including the attenuation of memory consolidation and retrieval, facilitation of the extinction of an activated avoidance response, and decrease of passive avoidance behavior, would support its potential role in PTSD neurobiology. The availability of intranasal OT that has been preliminarily demonstrated to be effective in other psychiatric conditions requires it to be tested in large samples of PTSD, given its relative absence of significant side effects, except for a transient amnesia that would be beneficial just in PTSD. Therefore, OT seems to represent a worthy target to investigate the pathophysiology and/or develop future drugs to treat this condition [122] (Figure 3).

### 4.6. Neuropeptide Y

**Figure 4:**
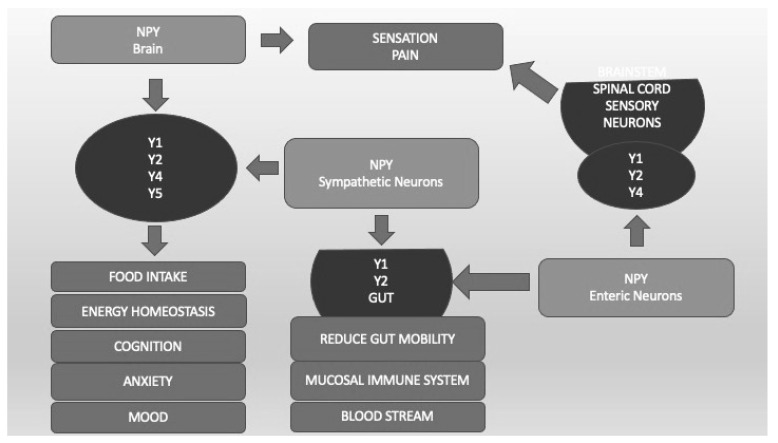
Neuropeptide Y (NPY) and its different receptors (Y1, Y2, Y4, Y5). Y1, Y2, Y4, and Y5, localized in different CNS areas and bound by NPY produced by CNS and sympathetic neurons, are important not only for food intake and energy homeostasis but also for cognition, anxiety, and mood regulation. Sensation and pain are regulated by CNS and enteric neurons that bind Y1, Y2, and Y4 localized in spinal cord sensory neurons. Y1 and Y2, located in the Gut system and bound by CNS and enteric NPY, regulate blood pressure, mucosal immunity, and gut mobility.

Neuropeptide Y (NPY) is a 36-amino-acid peptide that has widespread physiological and behavioral functions in the CNS and peripheral organs [123]. The physiological functions of NPY include the regulation of feeding behavior, energy homeostasis, blood pressure, reproductive behavior, and memory. Neuropeptide Y plays an important, albeit complex, role in regulating responses to stress and modulating fear and anxiety-related behaviors [123]. The different effects of NPY are mediated by at least five different G-protein-coupled receptors called Y1, Y2, Y4, Y5, and Y6 (Figure 4). Converging preclinical evidence supports NPY’s role in modulating the stress response and regulating anxiety in animal models. Advances in translational research point to NPY as a key mediator of stress response deserving attention for its potential therapeutic activity in PTSD. Therefore, both agonists of the Y2 and Y4 receptors appear to be potential novel treatments for anxiety disorders [124]. The combination of Y1 agonist and Y2 antagonist activity seems to reverse the alterations in learning and memory processes typical of PTSD [123]. All these findings are interesting but still preliminary and hypothetical (Figure 4).

### 4.7. MicroRNA

The microRNAs (miRNAs) are non-coding, single-stranded RNA molecules genomically encoded with 19–24 nucleotides that are coated with sequences complementary to messenger RNA (mRNA), thus regulating protein expression. Starting from the consideration that PTSD requires exposure to a traumatic event and that genes are sensitive to stress and trauma, epigenetic alterations have received attention as a possible mechanism for the development and persistence of PTSD [125,126,127,128,129]. Epigenetic modifications, including DNA methylation, histone modifications, and ncRNA, have been implicated in a number of complex diseases, such as cardiovascular disease, cancer, and neurological diseases [130]. Abnormalities in miRNA expression can fine-tune the expression of multiple genes within a biological network, suggesting that its dysregulation may be crucial for many of the observed molecular changes in the pathogenesis of PTSD. These findings provide evidence that miRNA not only plays a critical role in the pathophysiology of PTSD but can also open up new avenues for the development of diagnostic tools and therapeutic targets for the PTSD phenotype. Some studies on rat models of PTSD identified the expression of mir-34c in the hypothalamus as an important factor involved in susceptibility to the disorder and the family of miRNAs involved in heart diseases following PTSD [131]. Recent research on the role of miR-144 in the modulation of fear highlights this miRNA as a possible candidate biomarker for assessing the efficacy of treatment interventions [132]. Griggs and colleagues revealed that MiR-182 expression plays a functional role in the long-term consolidation of fear memory. The researchers discovered that miR-182 specifically targets actin remodeling genes that are essential for memory and synaptic structure. Based on these findings, it is possible to inhibit the consolidation of fear and so improve the relief of fear by therapeutically targeting miR-182 to raise its expression in the brain after a traumatic experience [133]. According to Chen et al., downregulating miR-153- 3p in the hippocampus of rats might lower apoptosis, boost the density of neuronal dendritic spines, and eventually reduce PTSD-like symptoms [134]. One of the most extensively researched miRNAs, miR-124, is also the most prevalent and enriched miRNA in the brain. Numerous pathogenic processes, including psychiatric diseases, are mediated by miR-124. The amount of miR-124-3p was shown to be downregulated in the hippocampus of rats who experienced single, sustained stress. MiR-124 overexpression may reduce PTSD-like symptoms [135]. Additionally, studies revealed that rats subjected to a single protracted stressor showed a down-regulation of their levels of miR-124 in the hippocampus, and that an increase in miR-124 levels might reduce PTSD-like symptoms [135]. Others evaluated the role of miRNA in immunological dysfunction associated with PTSD [136]. Given the association between miRNAs and several CNS-related disease processes, recent studies have proposed miRNA as a potential biomarker for the diagnosis, treatment, and prognosis of PTSD in veterans [50,137]. 

Given the paucity of human trials, animal models simulating pathophysiological features of these disorders, such as deficits in fear extinction, have significantly contributed to our understanding of the underlying processes involved in decreasing dysregulated fear. Future miRNA-based treatments for the management of fear-related illnesses, such as phobias and PTSD, hold the intriguing possibility of targeting such miRNAs [138]. 

### 4.8. Pipeline of Currently Studied Drugs

To date, a large proportion of ongoing trials involve different forms of psychotherapy, often implemented together with pharmacological interventions. Indeed, such an integrative approach is demonstrative of the intrinsic complexity of PTSD, which requires addressing both the psychological and biological facets of the disorder. In addition, there is a growing interest in exploring unconventional and innovative therapeutic modalities that, while being remarkably eclectic, perfectly illustrate the heterogeneity of contemporary research in this field. These range from paired vagus nerve stimulation and neurofeedback to hyperbaric oxygen therapy and translingual neurostimulation. The investigation of eye movement desensitization reprocessing therapy, deep brain stimulation (DBS), and equine assisted therapy (EAT) further exemplifies the breadth and depth of current research efforts. Remarkably, the scope of investigation extends beyond the conventional boundaries of medicine and into holistic practices, as exemplified by trials studying the effects of Sudarshan Kriya Yoga (SKY) in PTSD management. This intriguing blend of conventional and complementary approaches signifies a paradigm shift in our understanding and management of PTSD.

In any case, albeit far from being the sole current focus, pharmacotherapy undoubtedly remains a cornerstone in the management of PTSD. In such a context, one of the prevailing themes within the current clinical trial scene seems to be the repurposing of old, existing medical compounds. For instance, the San Francisco Veterans Affairs Medical Center [NCT03339258] has been investigating the therapeutic potential of doxazosin, an anti-hypertensive medication, in mitigating nightmares, sleep disturbances, and additional clinical symptoms in PTSD. This application has spurred further exploration, with Charité University [NCT05360953] concurrently probing the combined effect of clonidine, another anti-hypertensive drug, and doxazosin in ameliorating PTSD-related nightmares.

The exploration of existing drugs extends into the field of cannabinoids, with another study [NCT04448808] led by Charité University specifically focusing on dronabinol, a synthetic form of THC. The primary aim is to evaluate dronabinol’s efficacy in ameliorating PTSD-induced nightmares, a distressing symptom that significantly affects patients’ quality of life. The potential of dronabinol to reduce nightmares and other PTSD symptoms like hypervigilance and recurrent thoughts could offer a novel approach to PTSD treatment. Another intriguing direction lies in the repurposing of clonidine, an old anti-hypertensive medication typically prescribed for high blood pressure but also used off-label for certain psychiatric conditions, such as adult ADHD. Specifically, a trial [NCT04877093] spearheaded by the Minneapolis Veterans Affairs Health Care System aims to investigate the application of low-dose clonidine for PTSD in veterans, who often experience higher rates of PTSD due to exposure to combat-related trauma. This study also aims at exploring whether the combination of psychotherapy and low-dose clonidine might lead to a greater reduction in PTSD symptoms than psychotherapy alone, highlighting an integrative approach to PTSD treatment. 

Indeed, while repurposing existing drugs offers promising avenues for PTSD treatment, a concurrent surge in investigations into novel therapeutics signifies the growing emphasis on innovation in this field. One such venture is a phase 2b study [NCT04951076] examining the utilization of BNC210, an orally available negative allosteric modulator of the alpha-7 (α7) nicotinic acetylcholine receptor investigational drug. The primary objective of this study is to assess the efficacy of BNC210 in reducing the severity of PTSD symptoms as compared to a placebo. If successful, this could highlight BNC210 as a groundbreaking therapeutic intervention in the PTSD landscape. Echoing the trend of exploring novel compounds, other trials are underway to investigate the effects of BI 1358894 [NCT05103657] and JZP150 [NCT05178316] on PTSD symptoms. The study of BI 1358894, a novel and potent PDE9A inhibitor, aims to evaluate its safety, tolerability, and efficacy in adults with PTSD, potentially contributing to the development of new treatment paradigms. Concurrently, the trial involving JZP150, a selective and potent antagonist of GlyT1, seeks to elucidate its therapeutic potential in alleviating PTSD symptoms.

Even the modality of drug administration represents another area under scrutiny in the quest for effective PTSD treatment strategies. A study led by Tri-Service General Hospital [NCT05254405] is pioneering the use of intravenous brexanolone, a steroid originally approved for postpartum depression. Specifically, this clinical trial seeks to understand the possible benefits and challenges of intravenous administration for PTSD therapy while observing the drug’s safety and efficacy when administered in a controlled environment. In another noteworthy approach, the efficacy of stellate ganglion block (SGB) is under scrutiny in the NCT05391971 study. SGB, a procedure typically used for pain management, involves injecting local anesthetics (bupivacaine in this specific case) near the stellate ganglion, a collection of nerves in the neck region. This procedure has been proposed to offer potential relief from PTSD symptoms through its neuromodulatory effects. This trial represents just another example of how treatment strategies to date considered “unconventional” are in fact being explored to tackle PTSD, underpinning the growing diversification and sophistication of therapeutic options in this field.

As a matter of fact, the trajectory of pharmacotherapeutic research for PTSD will further expand to include an array of new potential medications, as seen in the most recent clinical trials. For instance, the NCT05401565 study is evaluating the therapeutic potential of balovaptan, a vasopressin 1a receptor antagonist. Of note, another distinctive investigation involves the antiviral combination of glecaprevir and pibrentasvir for PTSD treatment [NCT05446857]. Specifically, this study aims at examining the effects of these hepatitis C virus-targeting agents on PTSD, hypothesizing that the therapeutic action might extend beyond their original antiviral intent. The results may potentially expand our understanding of the psychoneuroimmunological factors at play in PTSD, offering novel avenues for therapeutic intervention.

Finally, the boundaries of PTSD pharmacotherapy are being pushed even further with the investigation of methylone [NCT05741710], a psychoactive substance structurally related to the ‘club drug’ MDMA, building upon previous findings that highlight the therapeutic potential of similar substances. This groundbreaking research could redefine our perceptions of the therapeutic utility of such psychoactive substances in the management of PTSD and other mental health disorders (Table 1).

Undeniably, the investigations identified through the comprehensive exploration of ongoing clinical trials reflect a holistic and innovative approach to combating PTSD. Firstly, it is crucial to note that these trials represent the current forefront of PTSD pharmacotherapy research. From the repurposing of drugs such as doxazosin, clonidine, and dronabinol to the exploration of novel compounds like BNC210, BI 1358894, JZP150, balovaptan, glecaprevir/pibrentasvir, and methylone, the PTSD pharmacotherapy pipeline is undeniably rich and diverse. Secondly, the exploration of different treatment modalities such as the intravenous administration of brexanolone and the use of stellate ganglion block with bupivacaine highlights the tireless pursuit of optimal treatment strategies to enhance the clinical management of PTSD.

A conspicuous aspect of the ongoing investigation remains the emphasis on nightmares and sleep disturbances. These are prominent symptoms of PTSD, and their repeated appearance in these trials underscores the importance of addressing these symptoms for comprehensive PTSD management. Indeed, this collective focus further reinforces the significance of a multi-modal treatment approach, recognizing the complex symptomatology of PTSD that extends beyond mere psychological manifestations. In the context of a global drug development perspective, these trials represent a concerted global effort in the quest for more effective pharmacological interventions for PTSD. Not only do they embody a dynamic and global endeavor to improve the lives of those living with PTSD, but they also serve to bridge the gap between our understanding of the mechanistic underpinnings of PTSD and the development of efficacious, target-oriented therapeutic strategies.

Indeed, these ongoing trials showcase the vibrant landscape of PTSD pharmacotherapy research and development. Their outcomes will invariably shape the future direction of PTSD treatment, helping to answer the critical question, “How close are we?” As researchers and clinicians await these findings, these trials underline the enduring commitment to optimizing therapeutic strategies for PTSD, bringing us ever closer to more effective and tailored treatment paradigms.

## 5. Conclusions

Post-traumatic stress disorder is a widespread and severe psychiatric disorder with a huge social and economic impact. The current guidelines include, as first-line treatments, SSRIs and SNRIs associated with CBT and EMDR, while some TCAs, mirtazapine, trazodone, and IMAOs (where available), anticonvulsants, and antipsychotics represent second-line strategies. However, it should be highlighted that the pharmacological strategies adopted to manage PTSD are largely empirical and mainly focused on symptom control. This attitude clearly reflects, on the one hand, the complexity of the clinical picture of PTSD, which is multifaceted, and, on the other, the lack of findings and even exhaustive hypotheses on the pathophysiology of this disorder. 

The HPA axis, glutamate, and cannabinoid systems, together with opioid peptides, OT, neuropeptide Y, and micro-RNAs, represent novel pathways and targets for potential interventions. Although most of the available data in humans are preliminary, some of them, such as those on cannabinoid modulators, is intriguing and at a more advanced stage than other similarly interesting compounds. The hope is that a closer link between basic research, animal findings, and controlled studies might lead to the rapid development of new and more tailored medications to be used for a broader range of PTSD symptoms. Indeed, given the recent past and current dramatic events (pandemics and post-pandemics, wars, immigration, economic recession, and climate change), there is no doubt there will be a larger part of populations worldwide exposed to threats and requiring specific and more effective treatments. 

## Figures and Tables

**Table 1:** Comprehensive overview of ongoing clinical trials focused on pipeline pharmacological interventions for PTSD as of July 2023.

Start Date	NCT Number	Status	Study Title	Interventions	Sponsor
November 2017	NCT03339258	Recruiting	A Randomized Controlled Trial of Doxazosin for Nightmares, Sleep Disturbance, and Non-Nightmare Clinical Symptoms in PTSD	Drug: Doxazosin Mesylate, Extended Release, Drug: Placebo	San Francisco Veterans Affairs Medical Center
June 2020	NCT04448808	Recruiting	Treating Nightmares in Posttraumatic Stress Disorder with Dronabinol	Drug: BX-1, Drug: Placebo	Charite University, Berlin, Germany
May 2021	NCT04877093	Recruiting	Repurposing Low-Dose Clonidine for PTSD in Veterans	Drug: Clonidine, Drug: Placebo	Aurora Health Care
July 2021	NCT04951076	Active, not recruiting	A Phase 2b Study of BNC210 Tablet Formulation in Adults with Post-Traumatic Stress Disorder (PTSD)	Drug: BNC210, Drug: Placebo	Bionomics Limited
November 2021	NCT05103657	Recruiting	A Study to Test Whether Taking BI 1358894 for 8 Weeks Helps Adults with Post-traumatic Stress Disorder	Drug: BI 1358894, Drug: Placebo	Boehringer Ingelheim
January 2022	NCT05178316	Recruiting	A Study of JZP150 in Adults with Posttraumatic Stress Disorder	Drug: JZP150, Drug: Placebo	Jazz Pharmaceutical
February 2022	NCT05254405	Recruiting	An Open Label Pilot Study of IV Brexanolone for the Treatment of Post-Traumatic Stress Disorder	Drug: Brexanolone Injection [Zulresso]	Donald Jeffrey Newport
May 2022	NCT05360953	Recruiting	Treating Nightmares in Posttraumatic Stress Disorder with Clonidine and Doxazosin	Drug: Clonidine, Drug: Doxazosin, Drug: Placebo	Charite University, Berlin, Germany
May 2022	NCT05391971	Recruiting	Effects of Stellate Ganglion Block in Post-traumatic Stress Disorder	Drug: Bupivacaine, Drug: Saline	NYU Langone Health
June 2022	NCT05401565	Recruiting	Study To Evaluate The Efficacy And Safety Of Balovaptan In Adults with Post-Traumatic Stress Disorder (PTSD)	Drug: Balovaptan, Drug: Placebo	Hoffmann-La Roche
July 2022	NCT05446857	Recruiting	Glecaprevir/Pibrentasvir for the Treatment of PTSD	Drug: Glecaprevir/Pibrentasvir Pill	White River Junction Veterans Affairs Medical Center
February 2023	NCT05741710	Recruiting	A Study to Assess the Use of Methylone in the Treatment of PTSD	Drug: Methylone, Drug: Placebo	Transcend Therapeutics

## Data Availability

Not applicable.
